# Editorial: Biotechnological applications of microbial strains from fermented foods

**DOI:** 10.3389/fmicb.2026.1953349

**Published:** 2026-08-25

**Authors:** Analía Graciela Abraham, Feng-Li Zhang, Joaquín Bautista-Gallego

**Affiliations:** 1 Centro de Investigación y Desarrollo en Ciencia y Tecnología de Alimentos (CIDCA, UNLP-CIC-CONICET) y Área Bioquímica y Control de Alimentos, Facultad de Ciencias Exactas, Universidad Nacional de La Plata (UNLP), La Plata, Argentina; 2 State Key Laboratory of Microbial Metabolism, School of Life Sciences and Biotechnology, Shanghai Jiao Tong University, Shanghai, China; 3 Departamento de Ciencias Biomédicas (Área de Microbiología), Facultad de Ciencias, Universidad de Extremadura, Badajoz, Spain

**Keywords:** bioengineering, biotechnology, fermentation technology, fermented foods, fermenting bacteria strains, food biotechnology, food safety, food technology

## Introduction

Fermented foods represent one of the richest reservoirs of microbial diversity encompassing bacteria, yeasts, and fungi that offer valuable resources for biotechnology and development of next-generation starter cultures, natural bioprotective agents, functional foods, and sustainable bioprocesses (Marco et al., 2021; Todorovic et al., 2024).

Furthermore, advances of genome sequencing, metagenomics, metabolomics, and predictive microbiology are transforming strain selection from taxonomic methods to evidence-based approaches, enabling tailored functionalities for food fermentation and health promotion. This transition toward evidence-based microbial biotechnology is enabling the rational development of microorganisms with tailored functionalities for food fermentation, health promotion, biomass valorization, and biological preservation (Leroy and De Vuyst, 2004; Hutkins, 2018).

The 14 articles in this Research Topic showcase the evolution of microbial biodiversity into biotechnology, demonstrating how microorganisms from fermented foods can enhance food quality, optimize fermentation, and improve food safety. Covering diverse microbial groups and food matrices, the collected studies converge toward a common objective: transforming the enormous biodiversity associated with fermented foods into innovative solutions for current and future challenges facing the agri-food sector.

We present an overview of these papers, which can be grouped under four research themes: (i) Identifying microbial resources from fermented foods and its application for biotransformation of functional foods and beverages (ii) optimization and control of fermentation processes; (iii) sustainable microbial biotechnology and circular bioeconomy; and (iv) novel strategies for food bioprotection.

## Identifying microbial resources from traditional fermented foods and its application for biotransformation of foods and beverages

The exploration of fermented foods has shifted from cataloging microbial diversity to identifying strain-specific functional phenotypes with technological relevance (Tamang et al., 2016; Augustin et al., 2024). The studies included in this Research Topic clearly illustrate this transition since highlights that traditional fermented foods, like Attiéké, artisanal raw-milk cheeses, kefir, Spanish-style table olives, and cured meats, are valuable sources of bacterial and yeast strains with complementary functional properties.

While microbial species are commonly regarded as functional units in food fermentation, industrial performance relies on physiological differences among strains. This concept is exemplified by the study of 66 *Lachancea thermotolerans* isolates from table olives. By combining predictive microbiology with multivariate statistical analyses, the authors revealed significant intraspecific variability in pH and salinity tolerance, as well as in L-lactic acid production (Gil-Flores et al.).

A similar strategy was applied to indigenous *Lactococcus lactis* strains from artisanal cheeses, where culture-dependent methods combined with shotgun metagenomics identified strains with desirable technological and functional traits (Nelon et al.). Likewise, whole-genome sequencing of *Lactococcus garvieae* ZB15 from traditional bacon confirmed its safety while supporting its probiotic and antifungal potential, highlighting the value of genomics for strain development (Li et al.).

The value of exploring traditional fermentations is further demonstrated by the identification of lactic acid bacteria from Attiéké displaying strong antimicrobial activity under elevated temperatures highlighting the need for resilient starter cultures in the face of climate change (Coulibaly et al.).

The shift in food microbial biotechnology focused on selecting microorganisms for specific traits, expanding the options for next-generation fermented foods and biotechnological applications, particularly in plant-based fermented products. Two contributions in this Research Topic demonstrate the potential of lactic acid bacteria demonstrate the potential of lactic acid bacteria as multifaceted starter cultures for cashew (Pietrantuono et al.) and quinoa-based (Fontana et al.) beverages, leading to rapid acidification, microbiological stability, and enhanced antioxidant properties. These findings highlight how carefully selected microorganisms can boost product stability, nutritional value, and health benefits, paving the way for innovative plant-based fermented foods.

Microbial biotransformation also enhances bioactive phytochemicals, as seen in the fermentation of Panax ginseng with *Aspergillus cristatus*, where genomics and metabolomics identified pathways converting ginsenosides to more active derivatives. Su et al. demonstrating how omics technologies exploit microbial enzymatic diversity for high-value products Additionally, in blueberry wine, the sequential inoculation of *Clavispora* sp. and *Saccharomyces cerevisiae* improved aroma and phenolic composition, showcasing how controlled microbial interactions create unique beverage profiles (Ma et al.).

These studies underscore a shift in food microbial biotechnology from discovering new microorganisms to selecting strains with specific functional traits, laying the groundwork for optimizing industrial fermentations.

## Optimization and control of fermentation processes

Modern fermentation science combines microbial physiology with predictive modeling to enhance industrial processes (McMeekin et al., 2008). This transition is illustrated by evaluating indigenous *Saccharomyces cerevisiae* strains for bioethanol production, where predictive microbiology assessed yeast responses to various conditions. Using mathematical growth models, researchers identified robust strains, showing that quantitative methods can speed up strain selection and reduce experimental screening from empirical process (Canseco Grellet et al.).

The review by Diao et al. offers a broader perspective on mustard fermentation, which highlights fermentation as a dynamic ecological process governed by complex microbial successions rather than by the activity of individual microorganisms. This review discusses how processing parameters influence microbial community assembly, metabolite production and flavor development, emphasizing the integration of metagenomics, metabolomics, synthetic biology, and precision fermentation as tools to rationally design safe starter cultures.

These contributions display a fundamental shift in contemporary fermentation biotechnology. By combining modeling, multi-omics, and controlled strategies, researchers can optimize microbes for specific goals, extending applications beyond food to sustainable uses.

## Sustainable microbial biotechnology and circular bioeconomy

The rising demand for sustainable food production has made fermentation essential for the circular bioeconomy, converting low-value agricultural materials into valuable products (Soccol et al., 2017).

This Research Topic includes several studies on biomass valorization strategies. For instance, a cold-adapted *Lactiplantibacillus plantarum* strain improved fermentation quality in Jerusalem artichoke silage on the Qinghai–Tibet Plateau (Wei et al.). Additionally, combining probiotic microorganisms with cellulolytic enzymes during corn stalk fermentation enhanced lignocellulose degradation and nutrient digestibility, thus boosting animal growth (Wei et al.). The concept of biomass valorization is further expanded by solid-state fermentation of corn husk for microbial protein production (Yan et al.) that underscores the role of microbial ecology in designing sustainable fermentation systems.

Overall, these studies highlight how microbial biotechnology contributes to sustainability beyond traditional food fermentation and is becoming an essential component of circular production systems.

## Novel strategies for food bioprotection

Food-associated microorganisms are increasingly viewed as sustainable alternatives to traditional preservation methods. Lactic acid bacteria are promising microorganisms due to their ability to produce a broad spectrum of antimicrobial compounds capable of inhibiting foodborne pathogens and spoilage microorganisms (Cotter et al., 2013; Gálvez et al., 2007). He et al. addressed a key challenge in protecting the bacteriocin-producing strain *Lactococcus lactis* LAB3 by encapsulating in calcium alginate-caseinate microbeads. Their research demonstrated that antimicrobial activity against *Listeria monocytogene*s depended on process variables. Importantly, the work moves beyond simply demonstrating antimicrobial potential *in vitro* by evaluating the stability and performance of the encapsulated culture under conditions relevant to commercial food preservation.

## Conclusions

All the studies gathered in this Research Topic collectively demonstrate that microbial strains associated with fermented foods have evolved from traditional fermentation agents into versatile platforms for modern biotechnology. Although covering diverse microbial groups, food matrices, and technological applications, all contributions converge toward a common objective: the identification, characterization, and rational exploitation of microbial functions to address emerging challenges in food production, sustainability, and human health.

Fermented foods will continue to represent not only a valuable part of the world's gastronomic heritage but also one of the most important reservoirs of microbial resources for developing the next generation of sustainable biotechnological innovations.
